# Editorial: Editors’ showcase 2022-2023: insights in nuclear organization and dynamics

**DOI:** 10.3389/fcell.2023.1340745

**Published:** 2023-12-06

**Authors:** Eric C. Schirmer

**Affiliations:** Institute of Cell Biology, University of Edinburgh, Edinburgh, United Kingdom

**Keywords:** Satb1, condensates, strutural variations, cancer, super resolution microscopy, nuclear pore complex (NPC), nucleo-cytoplasmic transport

The Section on Nuclear Organization and Dynamics has a wide range of expertise on its Editorial and Reviewer Boards and we have captured a snapshot of this in the set of papers highlighted in this Editor’s Showcase.

Zelenka et al. in previous studies of the T-cell CD4^+^/CD8^+^ stage of development had noted that the transcription factor SATB1 had additional functional roles (Cai et al., 2003; Feng et al., 2022; Zelenka et al., 2022). They postulated that these roles might be supported by another splice variant of SATB1 and in “A novel SATB1 protein isoform with different biophysical properties” they identified this novel variant. Apart from a beautiful characterization of this novel splice variant that included super-resolution microscopy imaging and identification of many interacting proteins, they found that it phase separates and does so in a manner regulated by phosphorylation. Notably, they also found it binds to non-coding RNAs and it has recently been demonstrated by Nobel Laureate Phil Sharp and others (Zhang et al., 2015; Sharp et al., 2022) that RNA binding can also drive phase separation. They demonstrated a propensity to phase separate for both the previous and novel isoforms, but further showed that an additional exon in the novel isoform also contained a prion-like domain that further enhances its phase-separation capabilities. In comparing ATAC-Seq cancer data, they found that the accessibility of the extra exon in the novel isoform (implying its expression) correlates with better outcomes in several cancers. Finally, consistent with splicing proteins being amongst its identified interacting proteins, the new SATB1 isoform seems to regulate its own splicing. These findings are important not just in their own right, but because after over a quarter century studying this transcription factor and massive amounts of high-throughput sequencing studies, a new splice variant can still be found. Editors at the Section on Nuclear Organization and Dynamics anticipate that there are thousands of as yet unidentified tissue- and developmental stage-specific splice variants and encourage papers identifying them as well as papers identifying new drivers of phase separation.

Stephenson-Gussinye and Furlan-Magaril presented an insightful overview of the evolving field using “Chromosome conformation capture technologies as tools to detect structural variations and their repercussion in chromatin 3D configuration.” Historically translocations were identified by chromosome spreads and subsequently through fusion points identified by genome sequencing; however, since 4C was first used on primary cancers in 2009 (Simonis et al., 2009), it has revolutionized the identification of these and other structural variations (SVs) by also revealing data about how the change affects regulation in adjacent regions, e.g., altering super-enhancer interactions that regulate expression of multiple genes that can contribute to the original cancer—for example, a gene hub supporting cell migration to drive metastasis. However, even less expensive techniques such as 3C can give much information about SVs in cancers that can inform on patient treatments and expected progression.

The labs of Hoboth et al. developed a way that the many millions of formalin fixed and paraffin embedded (FFPE) tissue sections can be used for quantitative multi-parameter investigations. In “Quantitative super-resolution microscopy reveals the differences in the nanoscale distribution of nuclear phosphatidylinositol 4,5-bisphosphate in human healthy skin and skin warts,” they developed protocols for using such samples to quantify nuclear phosphatidylinositol 4,5-bisphosphate (nPI(4,5)P2) levels within the nuclear speckle compartment. The authors had previously shown by that nPI(4,5)P2 levels are elevated in human papillomavirus (HPV)-associated cancer (Marx et al., 2018) and wondered if a staining protocol could be developed for use diagnostically as well as if it is similarly upregulated in HPV-induced warts that sometimes become malignant (Howley and Pfister, 2015). Stimulated emission depletion (STED) microscopy (Hell and Wichmann, 1994; Klar et al., 2000) is amongst the highest resolution super resolution microscopy approaches, while still being comparatively easy to use. They adapted a staining protocol for use with FFPE tissue sections to mark both nPI(4,5)P2 and nuclear speckles using STED. The paper is worth reading for the shear beauty of the staining alone, but they moreover demonstrated the increase in co-localization of the nPI(4,5)P2 and a nuclear speckle marker in HPV-induced warts compared to healthy skin. This suggests that similar markers could be used to distinguish disease samples and potentially even prognostically grade tumors.

Rush et al. presented a beautifully balanced overview of different models for nucleo-cytoplasmic transport in “Unveiling the complexity: assessing models describing the structure and function of the nuclear pore complex.” Notably, the beautiful historical overview highlights a number of misconceptions from oversimplification such as the typical textbook descriptions implying a rigid diffusion barrier when its nature is quite dynamic. This review is also very valuable in its accuracy and clear descriptions of the limitations of some of the techniques used to generate the data on which transport models are derived. This review is the most comprehensive I have encountered covering the Plug (Talcott and Moore, 1999), Polymer Brush (Rout et al., 2003), Oily Spaghetti (Macara, 2001), Hydrogel (Ribbeck and Gorlich, 2001), Reduction of Dimensionality (Peters, 2005), Forest (Yamada et al., 2010), Gradient (Ben-Efraim and Gerace, 2001), Dilation (Oberleithner et al., 2000), and Transport Receptor (Lim et al., 2006) models for central channel transport. In addition, mechanisms of transmembrane transport through the peripheral NPC channels are also described.

These papers highlight both the excellence and wide range of expertise among our editors at Frontiers. It should be noted that in addition to these great studies and reviews, Frontiers Nuclear Organization and Dynamics editors have contributed many excellent studies to many other Research Topics over the past year.
